# Triacylglyceride, Antioxidant and Antimicrobial Features of Virgin *Camellia* *oleifera*, *C. reticulata* and *C. sasanqua* Oils

**DOI:** 10.3390/molecules18044573

**Published:** 2013-04-18

**Authors:** Xesús Feás, Leticia M. Estevinho, Carmen Salinero, Pilar Vela, María J. Sainz, María Pilar Vázquez-Tato, Julio A. Seijas

**Affiliations:** 1 Department of Organic Chemistry, Faculty of Sciences, University of Santiago de Compostela, E-27080 Lugo, Spain; E-Mails: xesus.feas@usc.es (X.F.); pilar.vazquez.tato@usc.es (M.P.V.-T.); 2 CIMO-Mountain Research Center, Agricultural College of Bragança, Polytechnic Institute of Bragança, Campus Santa Apolónia, E 5301-855 Bragança, Portugal; E-Mail: leticia@ipb.pt; 3 Areeiro Phytopathological Station, Pontevedra Deputation, Subida a la Robleda s/n, E36153 Pontevedra, Spain; E-Mails: carmen.salinero@depo.es (C.S.); pilar.vela@depo.es (P.V.); 4 Department of Plant Production, Faculty of Veterinary Sciences, University of Santiago de Compostela, E-27002 Lugo, Spain; E-Mail: mj.sainz@usc.es

**Keywords:** antioxidant potential, antimicrobial activity, Camellia, oil, triacylglyceride

## Abstract

Virgin oils obtained from seeds of *Camellia oleifera* (CO), *Camellia reticulata* (CR) and *Camellia sasanqua* (CS) were studied for their triacylglyceride composition, antioxidant and antimicrobial activities. Levels of fatty acids determined by ^1^H-nuclear magnetic resonance analysis were similar to those reported for olive oils (82.30%–84.47%; 5.69%–7.78%; 0.26%–0.41% and 8.04%–11.2%, for oleic, linoleic, linolenic and saturated acids, respectively). The CR oil showed the best antioxidant potential in the three *in vitro* models tested. With regard to EC_50_ values (µg/mL), the order in DPPH radical-scavenging was CR (33.48) *<* CO (35.20) *<* CS (54.87). Effectiveness in reducing power was CR (2.81) *<* CO (3.09) *<* CS (5.32). IC_50_ for LPO inhibition were 0.37, 0.52 and 0.75 µg/mL for CR, CO and CS, respectively. All the oils showed antimicrobial activity, and exhibited different selectivity and MICs for each microorganism tested (*E. coli*, *B. cereus* and *C. albicans*). *B. cereus* was the less sensitive species (MIC: 52.083 ± 18.042 for CO; 41.667 ± 18.042 for CR; 104.167 ± 36.084 for CS mg/mL) and the *E. coli* was the most sensitive to camellia oil’s effect. The standard gentamicin presented higher MIC for *E. coli* (4.2) than the CR (MIC= 2.6) and CO (MIC = 3.9) oils.

## 1. Introduction

The genus *Camellia* (*Theaceae*) is native to East Asia and comprises more than 200 woody evergreen species [1]. Some species possess great economic value, particularly *C. sinensis* (the tea plant) which is grown commercially mainly in tropical and subtropical regions. Other species such as *C. japonica*, *C. reticulata* and *C. sasanqua* are cultivated in temperate regions worldwide as ornamentals. The seeds of *Camellia* species can be pressed to obtain high quality oils, some of which have been used for years in Asian cultures. The oil from *C. oleifera* (widely known as tea seed oil or tea oil) is used extensively in the Hunan Province in China for cooking, and *C.* *japonica* oil has a long history of traditional cosmetic usage in Japan as a protectant to maintain the health of skin and hair [2].

*Camellia* spp. have been used also in Oriental ethnomedicine and appear to be very promising leads for possible pharmaceutical exploitation since modern science has made it possible to specify their potential medical significance with antimicrobial [3], antioxidant [4,5], anti-allergic [6,7], antiviral [8,9] and skin healing properties [2]. However, there is relatively little information on the biological activity of the oils from *Camellia* species, except for tea oil [10,11,12,13,14]. The unsaturated fatty acid content in tea oil can reach as much as 90%, which is the highest amount so far reported for unsaturated fatty acids in edible oils. Furthermore, tea oil is rich in catechin, polyphenols, saponin and squalene, which have good whitening and antioxidation, anti-permeability, anti-inflammatory, analgesic and anticancer properties [15]. Moreover, *Camellia* oil is often the target for adulteration or mislabeling because it is a high priced product with high nutritional and medical values [16].

Galicia (NW Spain) has a strong and increasing presence in *Camellia* markets. Today, the bulk of the industry’s exports are as young plants for flowering and gardening purposes, but this is gradually increasing to provide a more diverse and value added portfolio, and in this context the production of *Camellia* oil appears as a new opportunity.

In the present work virgin *Camellia* oils produced in Galicia from seeds of *C. oleifera*, *C. reticulata* and *C. sasanqua* were studied for their: (a) triacylglyceride composition by ^1^H-nuclear magnetic resonance (^1^H-NMR) analysis, (b) antioxidant potential [by (i) 2,2-diphenyl-1-picrylhydrazyl (DPPH) radical, (ii) reducing power and (iii) *β*-carotene bleaching assays] and (c) antimicrobial activity (against clinically isolated strains of *Escherichia coli*, *Bacillus cereus* and *Candida albicans*). The information obtained from the present study can be used to evaluate the potential use of *Camellia* oil as food product for improving human health and nutrition, as well as for other uses such as cooking/salad oil, food ingredient and in several industrial applications.

## 2. Results and Discussion

### 2.1. Triacylglycerol Profile

Edible oils are mainly made up of TAG which comprises more than 95% to 99% of the total lipids present. Each type of oil has a different TAG profile which determines the nature of its physicochemical and nutritional properties, and also provides information on the quality of the oil. In recent years, both industry and consumers have shown an increased interest in oils’ compositions. However, simple identification and quantification of TAG is not sufficient, because of its dependence on many factors, such as number and position(s) of double bonds, type of fatty acid, chain lengths and their positions on the glycerol backbone [17]. 

**Figure 1:**
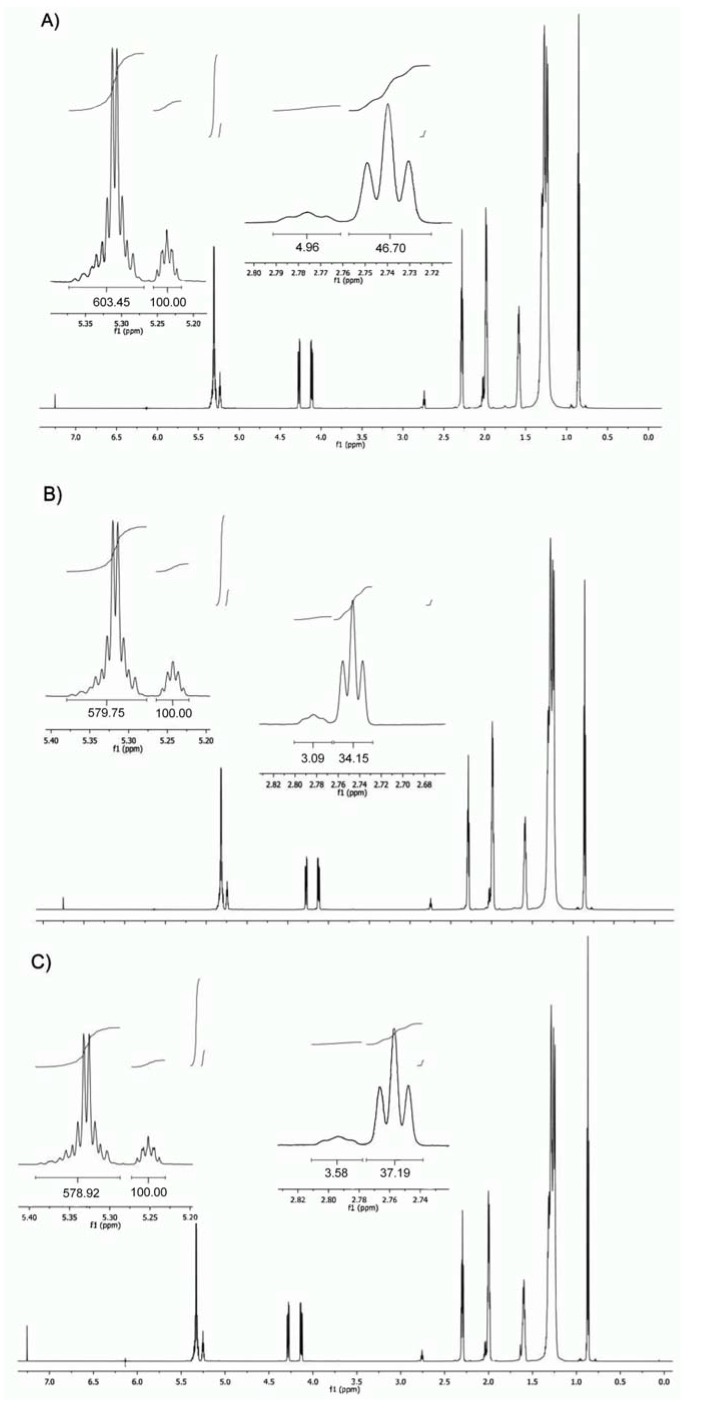
^1^H-NMR spectra (750 MHz for ^1^H) of *C. oleifera* (**A**), *C.* *reticulata*(**B**)and *C. sasanqua* (**C**) oils.

Figure 1 shows the expanded ^1^H-NMR spectrum of the analyzed *Camellia* oils. Vinylic hydrogens have a characteristic chemical shift, and could be used to determine the ratio of saturated to unsaturated esters. Bisallylic H could be used to differentiate the nature of the unsaturated components. Finally, the tertiary H in the glycerin moiety could be used to quantify the ratio of saturated to unsaturated esters since there is only one H for each TAG molecule. 1H-NMR has become one of the most promising methods to determine organic structures in complex matrices since do not depend on the efficiency of the sample treatment processes [18]. In ^1^H-NMR methodology, data is collected without sample pretreatment, thus rendering a simpler and faster analysis than the conventional methods. Moreover, it avoids several problems, such as lipid oxidation, involved in the traditional GC analysis and it does not require calibration with standards [19]. Furthermore, 1H-NMR offers advantages over HPLC or GC methodology because it allows the simultaneous, noninvasive, and nondestructive study of oil composition, and also provides information about the acyl position and distribution of TAGs [20,21,22,23,24]. However a magnetic field equal to 7.05 T (300 MHz) does not provide enough resolution to avoid signal overlapping between linoleic and linolenic acids. Magnetic fields equal to or higher than 9.4 T (400 MHz) are more appropriate [25], since a typical spectra obtained with 300 MHz spectrometer did not allow the accurate integration of the tertiary H of the glycerin moiety, and no difference was found between bisallylic H (δ = 2.80 ppm approx.) [26]. The use of a 750 MHz spectrometer to analyze the samples rendered a higher resolution spectra that in turn allowed for the separated integration of the signal from the tertiary glycerin H, and the vinylic ones.

Levels in percentage of C_18_ unsaturated [oleic acid (OA, C18:1n9c+t), linoleic acid (LA, C18:2n6c) and linolenic acid (LNA, C18:3n3)], the total saturated FA (SFA), monounsaturated FA (MUFA), polyunsaturated FA (PUFA), total unsaturated FA (TUFA = ∑MUFA + ∑PUFA) and the ratios PUFA/SFA, SFA/TUFA, and ω-6/ω-3, ω-3/ω-6, obtained for the virgin CO, CR and CS oil samples are shown in Table 1. The principal fatty acid found was oleic acid, ranging between 82.30% and 84.47%. It was followed by linoleic (5.69% to 7.78%) and linolenic (0.30 to 0.41). SFA and MUFA are synthesized endogenously in humans; however, PUFA needs to be supplied exogenously. The LA and LNA are key compounds for cell membranes and are associated to brain function and neurotransmission. These FA also play an important role in the transference of the O_2_ to blood plasma, in the synthesis of hemoglobin and in cellular division [27,28,29]. Moreover, FA from ω-6 series are biogenetic precursors of some physiologically important thromboxanes, leukotrienes and prostaglandins hormones, which are related to the inflammatory response. The nutritional value of essential ω-3 and ω-6 FA is also widely known for its health beneficial effects [30]. 

All the analyzed samples showed PUFA/SFA ratios above 0.58 and n-6/n-3 fatty acids of 0.05 (Table 1). PUFA/SFA ratios are used to calculate the risk factor of foods, since it is known that SFA increases the plasma cholesterol levels, while PUFA decreases them. 

**Table 1:** Levels (%) of fatty acid composition obtained for *Camellia* oils investigated.

**Oil**	**C18:1n9c+t**	**C18:2n6c**	**C18:3n3**	**∑SFA**	**∑MUFA**	**∑PUFA**	**∑TUFA**	**ω6/ω3**	**ω3/ω6**	**PUFA/SFA**	**TUFA/SFA**	**SFA/TUFA**	**Reference**
Virgin *C*. *oleifera*	83.77	7.78	0.41	8.04	83.77	8.19	91.96	0.05	18.98	1.02	11.44	0.09	PW *
Virgin *C*. *reticulata*	84.47	5.69	0.26	9.58	84.47	5.95	90.42	0.05	21.88	0.62	9.44	0.11	PW *
Virgin *C*. *sasanqua*	82.3	6.2	0.3	11.2	82.30	6.5	88.80	0.05	20.67	0.58	7.93	0.13	PW *
*C. japonica*	80.67	6.65	0.29	10.0	80.67	6.94	87.61	0.04	22.93	0.69	8.76	0.11	[21]
Virgin olive	80	5.9	0.7	13.4	80.00	6.60	86.60	0.12	8.43	0.49	6.46	0.15	[22]
Olive	77.5	7.4	0.7	14.4	77.50	8.10	85.60	0.09	10.57	0.56	5.94	0.17	[22]
Hazelnut	81	10.7	nd	8.3	81.00	10.70	91.70	0.00	-	1.29	11.05	0.09	[22]
Corn	33	51	0.7	15.3	33.00	51.70	84.70	0.01	72.86	3.38	5.54	0.18	[22]
Sunflower	29.2	58.8	nd	12	29.20	58.80	88.00	0.00	-	4.90	7.33	0.14	[22]
Linseed	20	17.1	54.2	8.7	20.00	71.30	91.30	3.17	0.32	8.20	10.49	0.10	[22]
Avocado	65	10	1	20	65.00	11.00	76.00	0.10	10.00	0.55	3.80	0.26	[23]
Tea seed	80	10	<1	10	80.00	-	-	-	-	-	-	-	[23]
Pumpkin	40.0	40.0	<1	10.0	40.00	-	-	-	-	-	-	-	[23]
Soybean	25.0	50.0	7.0	15.0	25.00	57.00	82.00	0.14	7.14	3.80	5.47	0.18	[23]
Canola	60.0	20.0	10.0	7.0	60.00	30.00	90.00	0.50	2.00	4.29	12.86	0.08	[23]

* Present work. Capric acid (C10:0); Palmitic acid (C16:0); Oleic acid (C18:1n9c+t); Linoleic acid (C18:2n6c); α-Linolenic acid (C18:3n3); Arachidic acid (C20:0); Eicosenoic acid (C20:1c). SFA: Saturated fatty acids (C10:0 + C16:0 + C20:0); MUFA: Monounsaturated fatty acids (C18:1n9c+t + C20:1c); PUFA: Polyunsaturated fatty acids (C18:2*n*6 + C18:3*n*3); TUFA: Total unsaturated fatty acids (∑MUFA +∑PUFA).

For comparative purposes, typical levels (in %) of C_18_ unsaturated FA, total saturated FA and ratios in common oils are shown in Table 1. *Camellia* oil has fatty acid composition similar to olive oil, in fact, it sometimes is referred to as the “olive oil of Asia” [10]. 

### 2.2. Antioxidant Activity

Results obtained for antioxidant activity of investigated *Camellia* oil samples are reported in the Table 2. In our research we used three established *in vitro* systems, since the antioxidant properties of food matrices cannot be evaluated by just one method due to the complex nature of their constituents. 

**Table 2:** Values (mg/mL) obtained in the antioxidant activity assays (EC_50_ for DPPH radical-scavenging activity and reducing power, and IC_50_ for inhibition of lipid peroxidation) of *Camellia* oils tested.

**Test**	***C. oleifera***	***C.reticulata***	***C.sasanqua***	**Control**
*DPPH scavenging*	35.20 ± 4.95 ab	33.48 ± 7.65 a	54.87 ± 8.78 c	47.02 ± 2.98 bc
*Reducing power*	3.09 ± 0.92 a	2.81 ± 0.63 a	5.32 ± 0.98 b	30.11 ± 1.67 c
*LPO inhibition*	0.52 ± 0.01 a	0.37 ± 0.01 a	0.75 ± 0.02 b	3.24 ± 0.56 c

In each column different letters mean significant differences (*p* < 0.05); Values for the standard Trolox in μg/mL.

The results obtained for the tested oils in each method used are shown below, with percent values in parenthesis. The results for CR and CO are similar, but superior in comparison with CS:

- DPPH scavenging activity (EC_50_) : CR (33.48) *<* CO (35.20) *<* CS (54.87)
- Reducing power (EC_50_): CR (2.81) < CO (3.09) < CS (5.32)
- LPO inhibition (IC_50_): CR (0.37) < CO (0.52) < CS (0.75)

Significant differences were found between the values obtained for the antioxidant activity of the different oils, using the three methodologies. The mean obtained for the oil of *C.* *sasanqua* was, in all cases, significantly different from the results obtained for *C. oleifera* and *C.* *reticulata.* The antioxidant potential of the control always differed significantly from the potential of the samples.

In the present work cold-pressed oil was employed since refined vegetable oils present reduced amounts of antioxidants as a consequence of the refining process [31]. Peroxidation of lipids is a process with important implications: it shortens the shelf-life of food and drugs, causes fragmentation of DNA, damages cellular membranes and promotes the genesis of many human diseases. Much effort is therefore devoted to search for “potent antioxidants”, both synthetic and from natural sources, particularly from plants [32]. Moreover, the crucial role of lipids in cell, tissue and organ physiology is demonstrated by genetic studies and human diseases that involve the disruption of lipid metabolic enzymes and pathways [18].

Extensive studies have been made about the total antioxidant capacity of edible vegetable oils, to prevent the presumed deleterious effects of free radicals in the human body, and to prevent the deterioration of fats and other constituents of foodstuffs. Nevertheless, to our knowledge there is scarce data reporting the antioxidant potential of *Camellia* oil and available works are focused on extracts, which involve the use of several organic solvents and not on whole oil material [11,13]. Based on the scavenging effects of different extracts of tea seed oil on DDPH radical it was suggested that apart from its traditional pharmacological effects, the tea seed oil may also act as a prophylactic agent to prevent free radical related diseases [33]. Zhang showed that the refined CO is capable to scavenge ·OH free radicals with an EC_50_ of 0.7165 µg/mL, equivalent to that of 5.31 µg/mL quercetin [27]. The methanol extract of CO exhibited pronounced radical scavenging activity against the stable DPPH radical (with antioxidant capacity EC_50_ value of 52.37 µg/mL). In fact, sesamin and 2,5-bis-benzo[1,3]dioxol-5-yl-tetrahydrofuro[3,4-d][1,3]dioxin were isolated from the methanol extract of CO and both compounds have remarkably protective effects against oxidative stress-related damages [33]. 

### 2.3. Antimicrobial Activity

Table 3 shows the antimicrobial activity screening of the three *Camellia* oils (*C. reticulata*, *C. oleifera* and *C. sasanqua*) on the bacterial strains (*B.* *cereus* – Gram positive and *E.* *coli* – Gram negative) and yeast (*C.* *albicans*). The MIC was used as a parameter of the significant inhibitory effects induced by oils on the growth of the tested microorganisms, as indicated by the TCC staining (dead cells are not stained by TTC). All the oils evidenced antimicrobial activity, and showed different selectivity and MICs for each microorganism. CR oil showed best antimicrobial activity in comparison with the other oils used for all microorganisms tested, followed by CO and CS.

**Table 3:** Minimum inhibitory concentration (MIC, in mg/mL) for the studied microorganisms in the antimicrobial activity assays with *Camellia* oils.

**MIC**	**C. *oleifera***	**C. *reticulata***	**C. *sasanqua***	**Control**
*B. cereus* (ESA 239)	52.083 ± 18.042 b	41.667 ± 18.042 b	104.167 ± 36.084 c	5.08 ± 0.35 a ^1^
*C. albicans* (ESA 567)	20.833 ± 7.217 ab	20.833 ± 7.217 ab	29.167 ± 19.094 b	0.65 ± 0.56 a ^2^
*E. coli* (ESA 34)	3.917 ± 3.406 ab	2.600 ± 1.125 ab	5.883 ± 3.406 b	4.22 ± 1.32 a ^1^

In each column different letters mean significant differences (*p* < 0.05). ^1^ Gentamicin and ^2^ amphotericin B in μg/mL.

The treatment of bacterial infections is increasingly complicated by the ability of the bacteria to develop resistance to antimicrobial agents [34]. Numerous reports have emphasized the need for less and better use of antibacterials, improved infection control, and the development of new agents [35]. Some of the common nosocomial infections, caused by *B.* *cereus*, *E.* *coli* and *C.* *albicans*, are urinary tract infections, respiratory pneumonia, surgical site wound infections, bacteremia, gastrointestinal and skin infections.

*B.* *cereus* was the less sensitive species to the *Camellia* oils’ effect (MIC: 52.083 ± 18.042 mg/mL for CO; 41.667 ± 18.042 mg/mL for CR; 104.167 ± 36.084 mg/mL for CS). *B. cereus* is a widely distributed foodborne pathogen that causes vomiting and diarrhea in mammals, including humans. Interest in *B.* *cereus* has been growing lately because it seems that *B.* *cereus*-related diseases, in particular food poisonings, are growing in number [36]. Although *B.* *cereus* is associated mainly with food poisoning, it has been increasingly reported to be a cause of serious and potentially fatal non-gastrointestinal-tract infections [37]. 

Antimicrobial activity of the tested *Camellia* oils against *C.* *albicans* was more homogenous, the obtained MICs ranging between 20.833 ± 7.217 mg/mL (for CO and CR) to 29.167 ± 19.094 mg/mL for CS. The incidence of fungal infections is increasing in community and hospital environments [38], and no other mycotic pathogen produces such a spectrum of opportunistic diseases in humans and animals as *Candida* does [39]. As it is very difficult to eliminate these microorganisms, due to their resistance to most antimicrobial agents, we decided to test the effect of camellia oil against them.

*E. coli* was the most sensitive to the *Camellia* oil’s effect, showing the control used (gentamicin) higher MIC (4.2) than the CR (MIC = 2.6) and CO (MIC = 3.9) oils. *E. coli* is a highly adapted pathogen capable of causing a range of diseases, from gastroenteritis to extraintestinal infections of the urinary tract, bloodstream and central nervous system [40]. The worldwide burden of these diseases is staggering, with hundreds of millions of people affected annually [41]. Moreover, *E. coli* has shown some resistance to gentamicin [42]. Recently, it was found that oil tea saponin extracted from the residual oil tea seed cakes had a remarkably restraining effect on the bacteria *E*. *coli*, *Staphylococcus aureus*, and *Bacillus subtilis* and on the fungi *Mucor racemosus*, *Aspergillus oryzae*, *Rhizophus stolonifer*, *Saccharomyces cerevisiae* and *Penicillium glaucum* [43,44].

Significant differences were found between the antimicrobial activity of the control and the obtained for the oils. Similarly to the observed concerning the antioxidant activity, the antimicrobial activity of the *C.* *sasanqua* oil was, in all cases, significantly different from the obtained for *C. oleifera* and *C*. *reticulata.*

Generally it is shown that Gram (−) bacteria are more resistant to plant based antimicrobials than Gram (+) bacteria. This may be explained by the structural differences of the bacterial cell wall. Gram (−) bacteria, apart from the cell membrane, possess an additional outer layer membrane, which consists of phospholipids, proteins and lipopolysaccharides, and this membrane is impermeable to most molecules. However, our results indicate that the tested *Camellia* oils can outcome in greater growth inhibition of Gram (−) bacteria than Gram (+). These findings agree with results from other studies about antimicrobial activity of saponin-rich fraction from *Camellia* *oleifera* cake [45] and the proanthocyanidin fraction separated from the crude *Camellia* tea extract [46]. However, the mechanisms of action of the antibacterial activity of saponins against both Gram (−) and Gram (+) bacteria are not yet clear [42,47].

## 3. Experimental

### 3.1. Chemicals and Reagents

2,2-Diphenyl-1-picryl-hydrazyl (DPPH) was obtained from Alfa Aesar (Ward Hill, MA, USA). 2,3,5-triphenyl-2H-tetrazolium chloride (TTC), linoleic acid (LA), polyoxyethylene (20) sorbitan monooleate (Tween 80), β-carotene, dimethyl sulfoxide (DMSO), ethyl acetate (EtOAc), trichloroacetic acid (TCA), 6-hydroxy-2,5,7,8-tetramethylchroman-2-carboxylic acid (Trolox^®^), iron(III) chloride (FeCl_3_), sodium dihydrogen phosphate (NaH_2_PO_4_), disodium hydrogen phosphate (Na_2_HPO_4_), potassium ferricyanide (III) [K_3_Fe(CN)_6_] and deuterated chloroform (CDCl_3_) were obtained from Sigma Chemical Co. (St. Louis, MO, USA). Trichloromethane (TCM), gentamicin and amphotericin B were obtained from Merck (Darmstadt, Germany). Methanol (MeOH) was obtained from Pronolab (Lisboa, Portugal). High purity and double distilled water (18 MX cm), which was used in all experiments, was obtained from a Milli-Q purification system (Millipore, Bedford, MA, USA).

### 3.2. Apparatus

Spectrophotometric measurements were made using a Unicam Helios AlphaUV-visible spectrometer (Thermo Spectronic, Cambridge, UK). ^1^H-NMR analyses were performed on a Varian Inova 750 (750 MHz for ^1^H) instrument (Agilent Technologies^®^, Palo Alto, CA, USA), equipped with a 5 mm probe. Evaporation of organic solvents was performed with a rotavapor system, consisting of a rotary vacuum evaporator (Heidolph VV. 2000, Leuven, Belgium) with a water bath and a B169 vacuum pump (Buchi, Flawil, Switzerland). An Eppendorf^®^ (Hamburg, Germany) model 5810 R centrifuge was used.

### 3.3. Plant Material and Oil Extraction

Samples of fruits of three *Camellia* species: *C. oleifera* (CO), *C. reticulata* (CR) and *C. sasanqua* (CS) were collected in autumn 2011 from one healthy plant of each *Camellia* species grown in the live germplasm *Camellia* bank at the Estación Fitopatolóxica do Areeiro (Pontevedra, Galicia, NW Spain). The harvest was carried out when fruits began to split open and the seeds were visible, a phenological stage of fruit development that corresponds to the BBCH stage 88 described for *Camellia japonica* [48]. For each plant, a seed sample of 1 kg was taken and divided into five 200 g subsamples. Oil extraction was performed for each subsample. After cold-pressing, the yield of *Camellia* oil obtained for each species was: 24.8% for CO, 19.1% for CR, and 17.6% for CS.

### 3.4. ^1^H-NMR Analysis

For triacylglycerols (TAG) analysis, each oil sample, weighing 0.2 g, was dissolved in 400 µL of CDCl_3_ shaken in a vortex mixer, and the resulting mixture was placed into a 5-mm diameter ultra-precision nuclear magnetic resonance (NMR) sample tube. The temperature of the sample in the probe was 30 °C. The chemical shifts are reported in ppm, using the solvent proton signal as standard. The proton resonances of the TAG acyl chains were assigned as previously described [26] and are shown in Table 4. In order to increase the accuracy of the signal areas in representing the H amounts, the longitudinal relaxation time (*T_1_*) was determined by executing the pulse sequence inversion recovery [25]. The area of the signals was determined by using the software equipment, and the integrations were carried out three times to obtain average values. All figures of the ^1^H-NMR spectra and of the expanded ^1^H-NMR spectrum regions were plotted at a fixed value of absolute intensity to be valid for comparative purposes.

### 3.5. Antioxidant Activity

The oils were evaluated for antioxidant potential, after diluting aliquots in EtOAc [47], through *in vitro* model systems such as 1,1-diphenyl-2-picrylhydrazyl (DPPH) [49], β-carotene-linoleate model system (antioxidant activity-AA) and iron reducing capacity (reducing power) [50]. Trolox^®^ was used as standard, its concentration ranged from 2.5 to 50 μg/mL, depending on the methodology used. The EC_50_ value represents the concentration of the *Camellia* oil causing 50% inhibition in each assay carried out. 

**Table 4:** Assignment of the signals of *Camellia* oils ^1^H-NMR spectra (750 MHz for ^1^H).

**Signal**	**Functional group**	**Multiplicity**	**Chemical shift (ppm)**
**1**	I (t) –CH_3_	t	0.89–0.86
**2**	H (m) –CH_2_-	m	1.35–1.23
**3**	G (m) –CH_2_–C–CO_2_–	m	1.64–1.57
**4**	D (m) –CH_2_–CO_2_-	m	2.02–1.98
**5**	E (m) –CH_2_–CO_2_–	m	2.06–2.02
**6**	F (dt) –C–CH_2_–C=C–	dt	2.33–2.28
**7**	C (t) –C=C–CH_2_–C=C–	t	2.78–2.74
**8**	L (m) –C=C–CH_2_–C=C–CH_2_–C=C	m	2.81–2.78
**9**	A (dd) –C–CH_2_–O–CO–C	dd	4.15–4.06
**10**	B (dd) –C–CH_2_–O–CO–C	dd	4.30–4.26
**11**	K (m) CH(–C–O–CO–C–)_2_	m	5.27–5.24
**12**	J (m) C–HC=CH–C	m	5.37–5.30

Signal multiplicity: s, singlet; d, doublet; t, triplet; m, multiplet; dt, doublet of triplets; dd, doublet of doublets. The signal number agrees with those in Figure 1.

#### 3.5.1. DPPH Scavenging Activity

Various concentrations of oil (300 μL) were mixed with MeOH solution (2.7 mL) containing DPPH• (6 × 10^−5^ mol/L). The mixtures were shaken vigorously and left to stand for 60 min in the dark (until stable absorption values were obtained). The reduction of the DPPH• was measured by continuous monitoring and observing the decrease of absorption at 517 nm. The radical-scavenging activity (RSA) was calculated as a percentage of DPPH discoloration using the equation: %RSA = [(A_DPPH_ − A_S_)/A_DPPH_] × 100; where A_S_ is the absorbance of the solution when the oil has been added at a particular level and A_DPPH_ is the absorbance of the DPPH solution. 

#### 3.5.2. Reducing Power Assay

The oil (2.5 mL) was mixed with 0.2 M Na_2_HPO_4_–NaH_2_PO_4_ buffer (2.5 mL, pH 6.6) and 10 mg/mL K_3_Fe(CN)_6_ (2.5 mL). The mixture was incubated at 50 °C for 20 min. After 100 mg/mL TCA (2.5 mL) was added, the mixture was centrifuged at 650 g for 10 min. The upper layer (2.5 mL) was mixed with H_2_O (2.5 mL) and 1.0 mg/mL FeCl_3_ (0.5 mL). The mixture absorbance was measured at 700 nm. Oil concentration providing 0.5 of absorbance (EC_50_) was calculated from the graph of absorbance against oil concentration in the solution.

#### 3.5.3. β-Carotene Bleaching (BCB) Assay

A solution was prepared by dissolving β-carotene (2 mg) in TCM (10 mL). Afterwards the aforesaid solution (2 mL) was pipetted into a 200 mL round-bottom flask, and then the organic solvent was removed at 40 °C under vacuum. LA (40 mg), Tween 80 emulsifier (400 mg) and distilled H_2_O (100 mL) were added to the flask. The mixture was shaken and a portion of this emulsion (4.8 mL) was transferred into different test tubes containing different oil concentrations (200 μL). The tubes were shaken and incubated at 50 °C in a water bath. As soon as the emulsion was added to each tube, the zero time absorbance was measured at 470 nm. Absorbance readings were then recorded at 20-min intervals until the control sample had changed colour. A blank, devoid of β-carotene, was prepared for background subtraction. Lipid peroxidation inhibition (LPO) was calculated using the following equation: LPO = (β-carotene content after 2 h of assay/initial β-carotene content) × 100.

### 3.6. Antimicrobial Activity Tests

#### 3.6.1. Microbial Strains

The microorganisms used in this study were clinical strains of *Bacillus* *cereus* (ESA 239, from feces), *Candida* *albicans* (ESA 567, from mouth) and *Escherichia* *coli* (ESA 34, from urine), isolated in the Northeastern Hospital Center of Bragança (Portugal), and identified, using molecular biology techniques, in the Microbiology Laboratory of the Escola Superior Agraria (ESA) of Bragança.

#### 3.6.2. Growth Conditions

Strains were stored in Muller–Hinton medium plus 20% glycerol at −70 °C. Before experimental use, cultures from solid medium were subcultivated in liquid media, Nutrient Broth for bacteria and Yeast Peptone Agar for yeasts, incubated overnight and used as the source of inoculums for each experiment. The inoculum for the assays were prepared by diluting cell mass in 0.85% NaCl solution, adjusted to 0.5 MacFarland scale, confirmed by spectrophotometrical reading at 580 nm for *B. cereus* and *E. coli*, and 640 nm for *C. albicans*. Cell suspensions were finally diluted to 10^4^ CFU/mL in order to use them in the activity assays.

#### 3.6.3. Antimicrobial Assay

Antimicrobial tests were carried out according to [51], using Nutrient Broth or Yeasts Peptone Dextrose on microplate (96 wells). Oil was diluted in DMSO and transferred into the first draw-well, and serial dilutions were performed. The inoculum was added to all wells and the plates were incubated at 37 °C for 24 h (*B. cereus* and *E. coli*) and 25 °C for 48 h (*C. albicans*). Amphotericin B and gentamicin were used as controls. In each experiment a positive control (inoculated medium) and a negative control (medium) and DMSO control (DMSO with inoculated medium) was introduced. Antimicrobial activity was detected by adding 20 µL of 0.5% TTC solution. The Minimum Inhibitory Concentration (MIC) was defined as the lowest concentration of *Camellia* oil that inhibited visible growth. Three independent repetitions of each oil sample were performed.

### 3.7. Statistical Analysis

All the experiments were performed in triplicate (n = 3) and the results were expressed as mean ± standard deviation. The studies were conducted in a fully randomized manner and all the obtained data were tested regarding normal distribution (Shapiro-Wilk test) and homogeneity of variances (Levene and Brown-Forsythe tests). When these conditions were met, statistical analyses were performed using the One-dimensional Variance Analysis (One-way ANOVA) followed by a Tukey test. 

## 4. Conclusions

This study provides the first data on triacylglyceride, antioxidant and antibacterial activities of virgin *Camellia oleifera*, *C.* *reticulata* and *C.* *sasanqua* oils. 750 MHz ^1^H-NMR spectroscopy has proven to be a useful tool for the direct analysis of the triacylglyceride composition of these oils, their levels of FA being similar to those reported for olive oils. The data obtained clearly show the antioxidant and antimicrobial activities of the three oils, being *E.* *coli* the most sensitive to the *Camellia* oil’s inhibitory growth effect. This finding opens new possibilities for future therapeutic applications of the analyzed *Camellia* oils, although further studies are needed to evaluate their potential *in vivo* use.
